# THE ASSOCIATION BETWEEN SENSE OF PURPOSE IN LIFE AND REPEATED MEASURES OF HEALTH BEHAVIORS OVER TIME

**DOI:** 10.1093/geroni/igz038.2997

**Published:** 2019-11-08

**Authors:** Eric S Kim, Koichiro Shiba, Laura Kubzansky

**Affiliations:** 1 Harvard T.H. Chan School of Public Health, Boston, United States; 2 Harvard T.H. Chan School of Public Health, Boston, Massachusetts, United States

## Abstract

Although a stronger sense of purpose in life has been associated with reduced risk of chronic conditions and mortality, potential pathways underlying these associations remain understudied. In the present study, we tested if a higher baseline sense of purpose in life was associated with maintenance of recommended levels of five health behaviors. Prospective data included 13,771 adults from the Health and Retirement Study, who were assessed up to six times across an average of 9 years. In mixed models that adjusted for sociodemographic factors, those in the highest quartile of purpose, compared to those in the lowest quartile, had a higher likelihood of not smoking (RR=1.04; 95% CI: 1.03–1.06), remaining physically active (RR=1.42; 95% CI: 1.33–1.51), drinking moderate amounts of alcohol (RR=1.21; 95% CI: 1.13–1.29), not suffering from sleep problems (RR=1.29; 95% CI: 1.24–1.34), and maintaining healthy BMI (RR=1.09; 95% CI: 1.06–1.11) over follow-up.

